# Early Detection and Serial Monitoring of Anthracycline-Induced Cardiotoxicity Using T1-mapping Cardiac Magnetic Resonance Imaging: An Animal Study

**DOI:** 10.1038/s41598-017-02627-x

**Published:** 2017-06-01

**Authors:** Yoo Jin Hong, Heae Surng Park, Jeffrey Kihyun Park, Kyunghwa Han, Chul Hwan Park, Tai Kyung Kim, Sae Jong Yoo, Ji Yeon Lee, Pan Ki Kim, Jin Hur, Hye-Jeong Lee, Young Jin Kim, Young Joo Suh, Mun Young Paek, Byoung Wook Choi

**Affiliations:** 1 Department of Radiology and Research Institute of Radiological Science, Severance Hospital, Yonsei University Medical Centre, Seoul, South Korea; 2 Department of Pathology, Gangnam Severance Hospital, Yonsei University College of Medicine, Seoul, South Korea; 3 Department of Radiology and Research Institute of Radiological Science, Gangnam Severance Hospital, Yonsei University Medical Centre, Seoul, South Korea; 4 Department of Veterinary Surgery, College of Veterinary Medicine, Konkuk University, Seoul, South Korea; 5 Siemens Ltd., Seoul, South Korea

**Keywords:** Diagnostic markers, Heart failure

## Abstract

A reliable, non-invasive diagnostic method is needed for early detection and serial monitoring of cardiotoxicity, a well-known side effect of chemotherapy. This study aimed to assess the feasibility of T1-mapping cardiac magnetic resonance imaging (CMR) for evaluating subclinical myocardial changes in a doxorubicin-induced cardiotoxicity rabbit model. Adult male New Zealand White rabbits were injected twice-weekly with doxorubicin and subjected to CMR on a clinical 3T MR system before and every 2–4 weeks post-drug administration. Native T1 and extracellular volume (ECV) values were measured at six mid-left ventricle (LV) and specific LV lesions. Histological assessments evaluated myocardial injury and fibrosis. Three pre-model and 11 post-model animals were included. Myocardial injury was observed from 3 weeks. Mean LV myocardium ECV values increased significantly from week 3 before LV ejection fraction decreases (week 6), and ECVs of the RV upper/lower insertion sites and papillary muscle exceeded those of the LV. The mean native T1 value in the mid-LV increased significantly increased from week 6, and LV myocardium ECV correlated strongly with the degree of fibrosis (r = 0.979, *p* < 0.001). Myocardial T1 mapping, particularly ECV values, reliably and non-invasively detected early cardiotoxicity, allowing serial monitoring of chemotherapy-induced cardiotoxicity.

## Introduction

Cardiotoxicity is a well-recognized adverse effect of chemotherapy^1, 2^. Notoriously, the anthracycline class of cytotoxic agents, which are highly effective against many cancers, can lead to irreversible myocardial damage^3^. In addition, considerable myocardial damage can occur below the known threshold level^3–5^. Accordingly, the early detection of myocardial dysfunction and prevention of overt heart failure are important^3^. Several guidelines and recommendations for cardiotoxicity monitoring have been published^6, 7^, including the American Heart Association (AHA)/American College of Cardiology (ACC) guidelines for anthracycline-induced cardiotoxicity, which recommend the use of radionuclide imaging, including multi-gated acquisition (MUGA) scans, and echocardiography for ejection fraction monitoring^8, 9^. However, no other imaging modalities have been specified for cardiac function monitoring during anthracycline therapy. Accordingly, a reliable, non-invasive method is needed.

Myocardial T1 mapping is a promising technique that could improve the detection of diffuse myocardial changes and quantitatively evaluate such changes using parameters such as T1 and extracellular volume (ECV) values. By measuring T1-mapping parameters, this novel technique confers the additional benefit of detecting myocardial changes caused by chemotherapy-induced cardiotoxicity at an earlier time point than would be available with other modalities^10, 11^. Accordingly, the purpose of this study was to assess the diagnostic value of T1-mapping cardiac magnetic resonance imaging (CMR) for evaluating early myocardial changes, to evaluate histopathology, and correlate the quantitative values obtained via CMR T1 mapping and histology of a rabbit model of cardiotoxicity.

## Results

**Figure 1:**
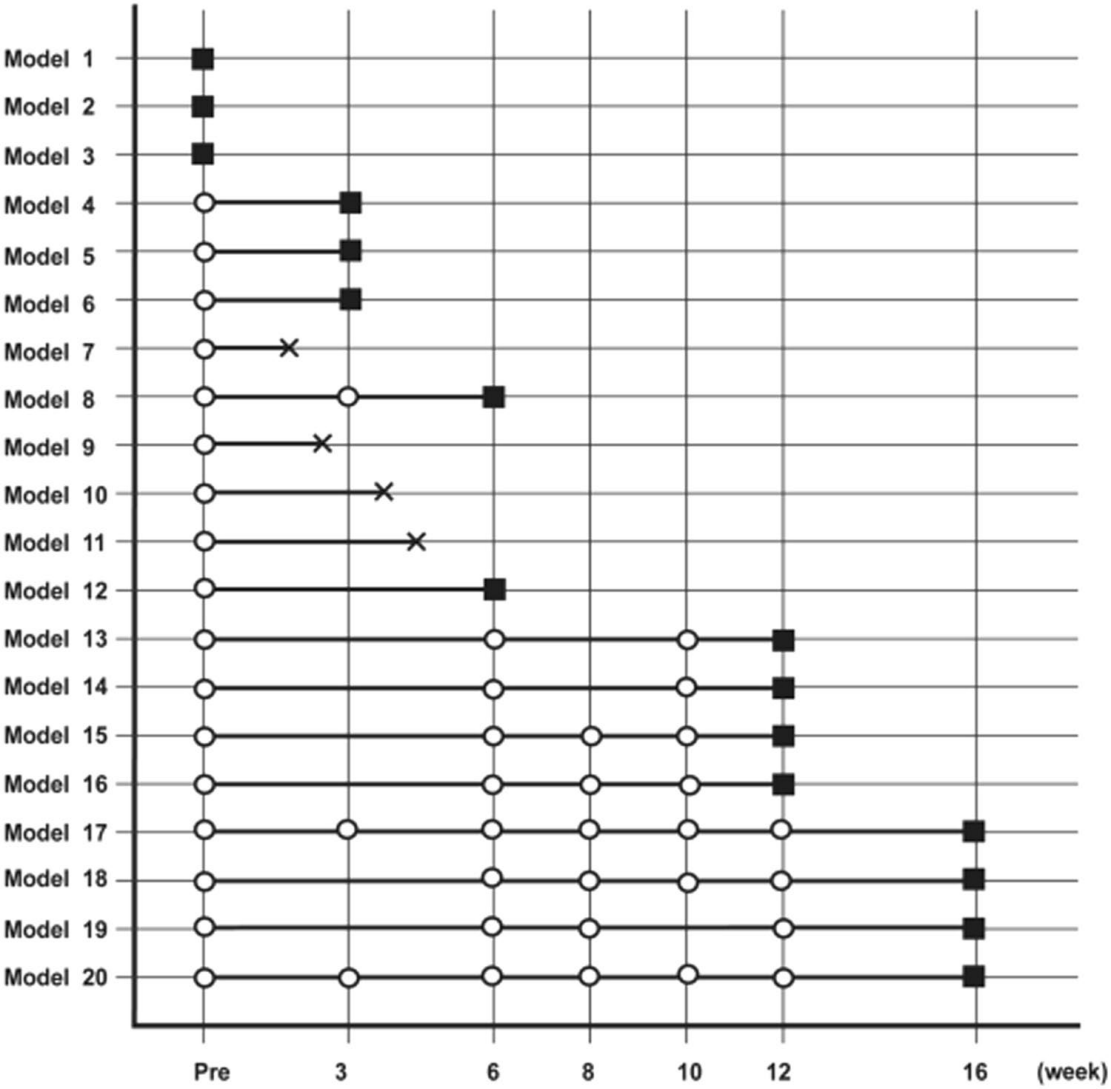
Experimental timeline and numbers of animals evaluated at each time point. All rabbits underwent cardiac magnetic resonance imaging (CMR) before and every 2–4 weeks after drug administration. Circles indicate scanning CMR; black squares indicate sacrifice after CMR scanning. Crosses indicate unexpected deaths.

A total of 20 rabbits were included in this study. After 3 weeks of modelling, three rabbits were sacrificed for histological evaluation after CMR scanning. The remaining rabbits remained subject to modelling and CMR scanning, and two and four additional rabbits were sacrificed for histological evaluation after 6 and 12 weeks, respectively. Modelling continued in the remaining rabbits, which were subjected to CMR scans until the end of the 16-week modelling period, followed by sacrifice for histological evaluation. We note that four rabbits died of severe anaemia (n = 1) or infection (n = 3) during the modelling period. Figure 1 presents the experimental timeline and numbers of animals evaluated at each time point.

### Physiological and functional data

Table 1 presents the physiological and functional data of the baseline and experimental modelling groups. The left ventricle ejection fraction (LVEF, %) decreased significantly beginning at week 6 of modelling. The mean LVEF of the initial baseline group was 56.08 ± 5.80%, compared to 44.59 ± 5.9% at week 6 of modelling (*p* < 0.001; Table 1). In addition to a decrease in left ventricle (LV) systolic function, a decrease in heart rate and morphologic LV changes, specifically chamber enlargement and wall thinning, were noted in the rabbit model of cardiotoxicity.

**Table 1:** Physiological and functional data of all subjects.

	Baseline subject	Week 3	Week 6	Week 8	Week 10	Week 12	Week 16
	(n = 16)	(n = 6)	(n = 10)	(n = 6)	(n = 7)	(n = 8)	(n = 4)
Weight (kg)	3.21 ± 0.40	3.19 ± 0.33	3.19 ± 0.34	3.53 ± 0.10	3.06 ± 0.58	3.18 ± 0.34	3.22 ± 0.46
Hematocrit (%)	41.69 ± 4.35	32.00 ± 4.75^*^	34.59 ± 5.64^*^	34.63 ± 5.08^*^	33.47 ± 3.35^*^	33.18 ± 5.98^*^	35.60 ± 8.58^*^
Heart rate (bpm/min)	177.15 ± 12.44	165.12 ± 25.95	164.39 ± 11.94	155.10 ± 13.34	154.41 ± 23.79	143.97 ± 20.74	155.98 ± 25.84
Stroke volume (mL)	1.34 ± 0.42	1.03 ± 0.44	1.13 ± 0.35	1.02 ± 0.19	1.35 ± 0.50	0.95 ± 0.42	0.95 ± 0.19
Left ventricle ejection fraction (%)	56.08 ± 5.8	52.67 ± 7.10	44.59 ± 5.9^*^	43.22 ± 4.10^*^	45.11 ± 6.20^*^	36.85 ± 9.38^*^	36.83 ± 2.34^*^
Cardiac output (mL/min)	237.84 ± 79.58	173.25 ± 90.00	186.85 ± 61.76	158.75 ± 37.41	204.31 ± 61.39	138.14 ± 60.18	155.98 ± 25.84
Left ventricle mass/weight (g/kg)	0.99 ± 0.24	0.98 ± 0.22	0.95 ± 0.17	0.90 ± 0.15	1.04 ± 0.30	0.92 ± 0.10	0.85 ± 0.83

^*^Indicated *p* < 0.05. All data are expressed as mean ± SD.

### T1 mapping CMR data

**Figure 2:**
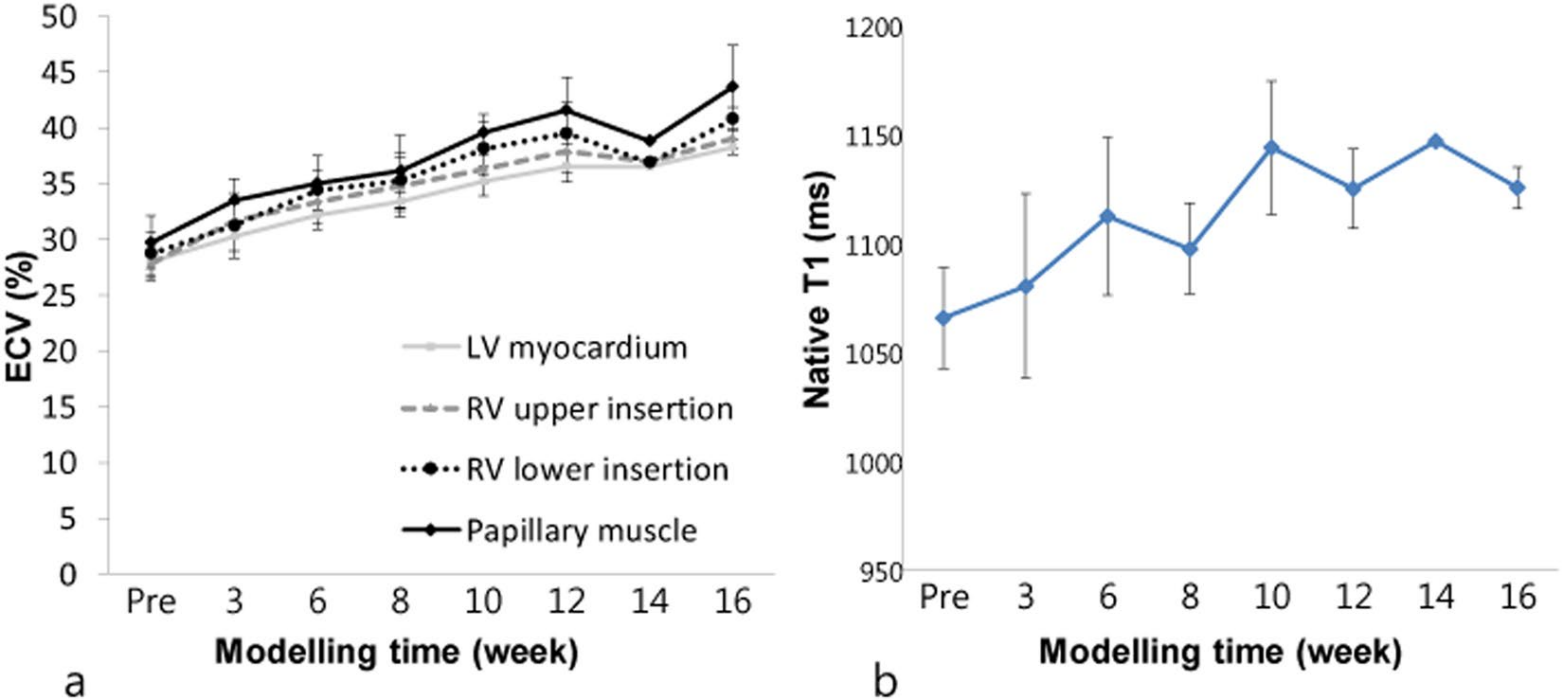
Serial changes in the extracellular volume (ECV) and native T1 values. (**a**) Serial changes in the ECVs of the left ventricle (LV) myocardium and other specific LV lesions according to modelling time. The mean ECV of the mid-LV increased significantly beginning at 3 weeks (*p* = 0.002). Compared with the LV myocardium, the ECVs of the right ventricle (RV) upper and lower insertion sites and the papillary muscle were higher. (**b**) Serial changes in the native T1 values of the LV myocardium according to modelling time. Mean native T1 values at the mid-LV increased significantly beginning at week 6 (*p* < 0.001).

The ECV and native T1 values changed as the modelling period increased. The mean ECV values of the mid-LV increased relative to those of the baseline group and significantly increased beginning at week 3 (*p* = 0.002; Table 2, Fig. 2a). Compared with the LV myocardium, the ECV values of the right ventricle (RV) upper/lower insertion sites and papillary muscle were higher than those of the LV myocardium. The mean native T1 values in the mid-LV also increased according to the modelling time. The native T1 value of the 6-week model differed significantly from the baseline (*p* < 0.001; Table 2 and Fig. 2b). The inter-observer agreements were good, with intra-class correlation coefficients (ICCs) (95% confidence interval [CI]) of 0.951 (0.919–0.971) for native T1 values, 0.958 (0.929–0.976) for the ECV of the LV myocardium, 0.915 (0.821–0.959) for the ECV of the RV upper insertion, 0.919 (0.830–0.961) for the ECV of the RV lower insertion, and 0.920 (0.832–0.962) for the ECV of the papillary muscle.

**Table 2:** Cardiac magnetic resonance (CMR) data of all subjects.

	Baseline subject	Week 3	Week 6	Week 8	Week 10	Week 12	Week 16
	(n = 16)	(n = 6)	(n = 10)	(n = 6)	(n = 7)	(n = 8)	(n = 4)
Native T1 value (whole myocardium) (ms)	1067.75 ± 24.08	1087.49 ± 46.13	1112.35 ± 38.36^*^	1097.48 ± 23.03^*^	1143.77 ± 33.29^*^	1124.92 ± 19.63^*^	1125.52 ± 10.82^*^
ECV (whole myocardium) (%)	27.95 ± 1.63	30.26 ± 1.28^*^	32.2 ± 1.29^*^	33.26 ± 0.84^*^	35.24 ± 1.45^*^	36.50 ± 1.36^*^	38.27 ± 0.68^*^
ECV (Upper RV insertion site) (%)	27.84 ± 1.25	31.69 ± 1.45^*^	33.36 ± 1.86^*^	34.77 ± 2.71^*^	36.26 ± 1.24^*^	37.91 ± 1.98^*^	39.09 ± 0.89^*^
ECV (Lower RV insertion site) (%)	28.73 ± 1.9	31.25 ± 2.91^*^	34.43 ± 1.78^*^	35.27 ± 2.48^*^	38.13 ± 2.43^*^	39.51 ± 2.89^*^	40.79 ± 0.98^*^
ECV (Papillary muscle) (%)	29.74 ± 2.37	33.48 ± 1.96^*^	35.01 ± 2.5^*^	36.11 ± 3.19^*^	39.55 ± 1.67^*^	41.61 ± 2.99^*^	43.69 ± 3.68^*^

### Histology correlation

**Figure 3:**
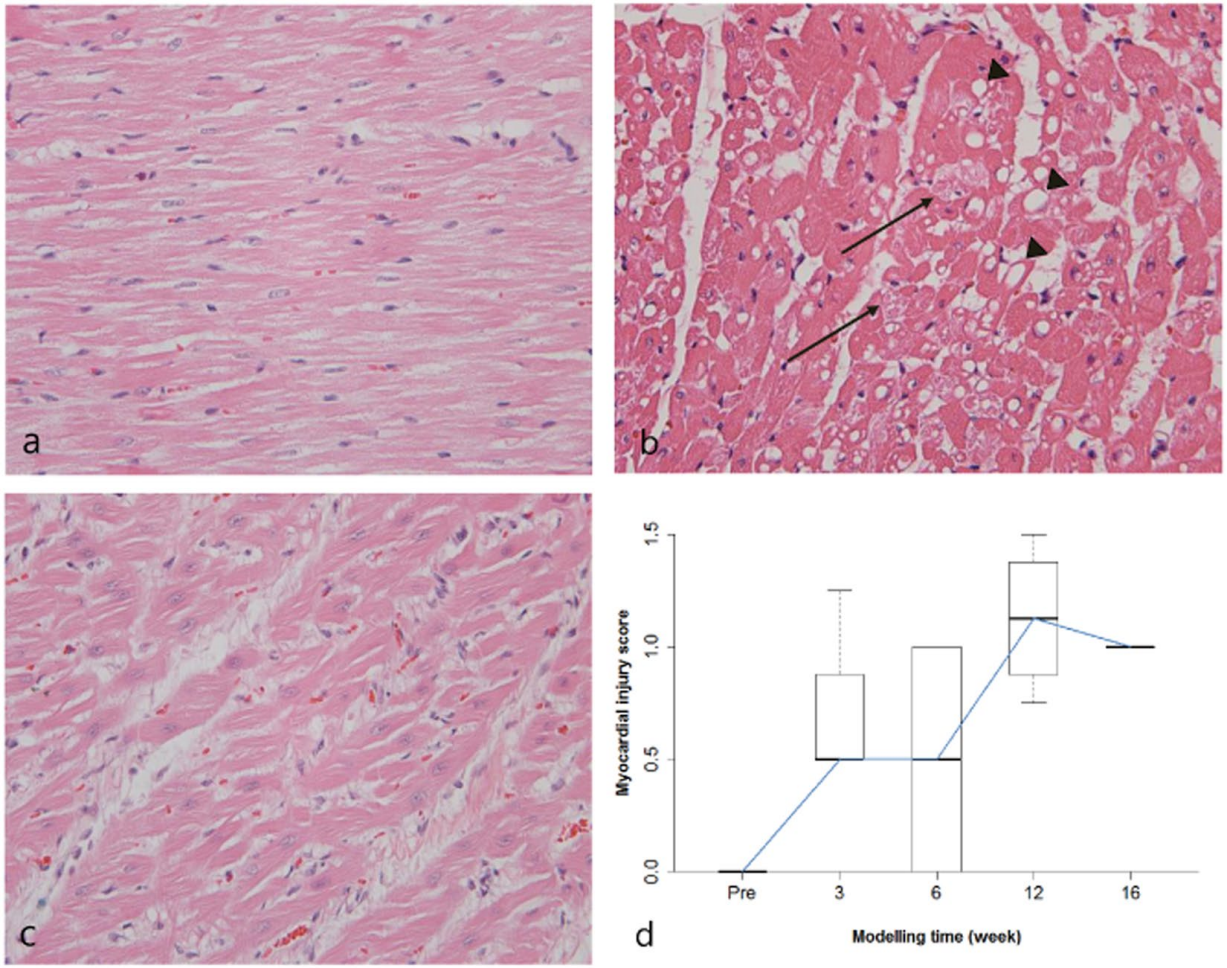
Histological findings of myocardial injury. (**a**) Normal myocardium from a control subject. (**b**) Severe myocardial injury with intracytoplasmic vacuolization (arrowheads) and myofibril loss (arrows) are visible in the left ventricular (LV) septal wall of a 3-week model (myocardial injury score = 3). (**c**) Diffuse interstitial oedema is visible in the LV lateral wall of a 3-week model. Haematoxylin and eosin staining; magnification, ×400 (**d**) Myocardial injury score according to the modelling time.

**Figure 4:**
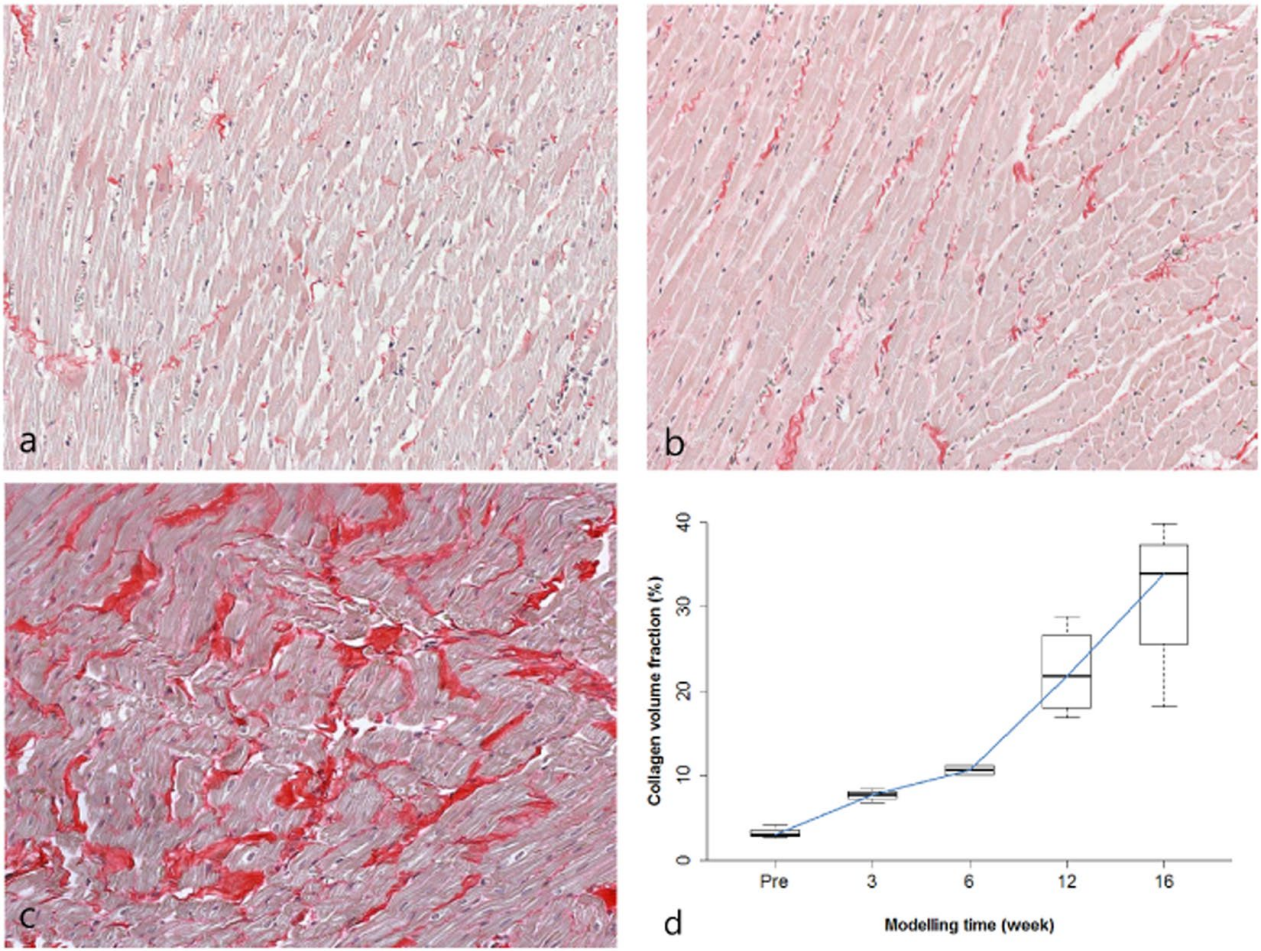
Histological images of extracellular collagen deposition in the left ventricle (LV) myocardium. (**a**) Minimal collagen fibres are visible in the interstitium of a baseline subject (measured collagen volume fraction [CVF] = 3.04%). (**b**) No significant increase in collagen fibres was observed in a 3-week model (CVF = 4.28%). (**c**) Abundant collagen deposition is visible in a 16-week model (CVF = 25.00%). Picrosirius red staining; original magnification, ×200 (**d**) Myocardial fibrosis according to the modelling time.

Myocardial injury was noted at an early modelling time point (week 3, Fig. 3b). Diffuse interstitial oedema was also noted in a 3-week model (Fig. 3c). The median myocardial score according to the modelling timeline is demonstrated in Table 3 and Fig. 3d. In baseline control subjects, microscopically, the LV myocardial interstitium contained minimal collagen fibres (Fig. 4a). Although even slight increases were not observed in the 3-week models (Fig. 4b), and in the 16-week model, abundant collagen deposition was observed in the myocardial interstitium (Fig. 4c). The median LV collagen volume fractions (CVFs) increased steadily according to modelling duration, and the mean CVFs at weeks 12 and 16 were significantly higher than the baseline (*p* = 0.020 and 0.003, respectively) (Table 3 and Fig. 4d). Although the ECV correlated positively with the histologic CVF (r = 0.979, *p* < 0.001), the native T1 value did not correlate with the histologic CVF (r = 0.455, *p* = 0.102). The RV insertion site had higher CVF values than the LV myocardium (*p* < 0.001), and the ECV of the RV insertion site correlated well with the CVF (r = 0.824, *p* < 0.001). Additionally, a correlation was observed between the myocardial injury score and degree of fibrosis according to the modelling time (r = 0.59, p = 0.02).

**Table 3:** Histologic data of all subjects.

	Baseline subject	Week 3	Week 6	Week 12	Week 16
	(n = 16)	(n = 6)	(n = 10)	(n = 8)	(n = 4)
CVF (%) [LV myocardium, median (range)]	3.04 (2.67–4.15)^†^	7.78 (6.86–8.47)^†^	10.67(10.14–11.21)^‡^	19.23 (16.87–28.84)^*,§^	33.17 (19.21–39.86)^*,§^
CVF (%) [RV insertion, median (range)]	5.69 (4.57–6.63)^†^	9.69 (9.65–10.01)^†^	15.72 (14.83–16.61)^‡^	29.44 (17.82–30.01)^*,§^	46.01 (18.71–51.76)^*,§^
Myocardial injury score [median (range)]	0 (0–0)	0.5 (0.5–1.25)^†^	0.5 (0–1)^‡^	1.125 (0.75–1.5)^§^	1.0 (1–1)^§^

^*^Indicated *p* < 0.05. ^†^Indicated n = 3, ^‡^indicated n = 2, ^§^indicated n = 4.

### Diagnostic value of T1 mapping parameters

**Figure 5:**
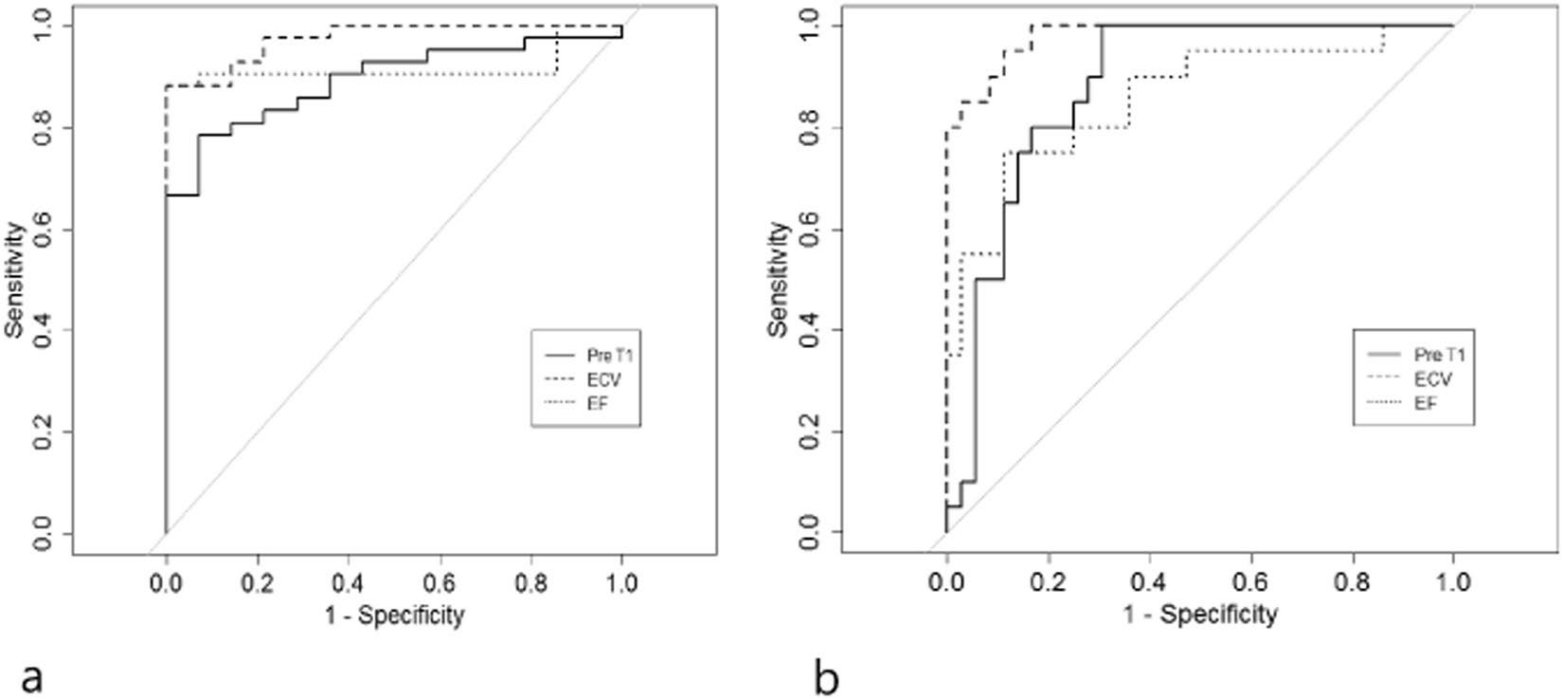
Receiver operating characteristic (ROC) curves for diagnosis. (**a**) Areas under the ROC curves (AUCs) for the extracellular volume (ECV), native T1, and left ventricle ejection fraction (LVEF) in post-modelling subjects were 0.974 (95% confidence interval, 0.955–0.989), 0.893 (0.848–0.930), and 0.916 (0.874–0.951), respectively. (**b**) In 12- and 16-week modelling subjects, the AUCs for the ECV, native T1, and LVEF were 0.981 (0.969–0.990), 0.882 (0.855–0.910), and 0.857 (0.801–0.906), respectively.

In the first analysis, post-modelling subjects (3-, 6-, 12-, 16-week models) were considered disease-positive and used to confirm the ability to detect early anthracycline-induced cardiotoxicity. The areas under the receiver operating characteristic (ROC) curves (AUCs) of the ECV, native T1, and LVEF were 0.974 (95% CI, 0.955–0.989), 0.893 (0.848–0.930), and 0.916 (0.874–0.951), respectively (Fig. 5a). The AUC of the ECV differed significantly from those of the ejection fraction (EF; *p = *0.001) and native T1 (*p* < 0.001), whereas the AUCs of the native T1 and EF did not differ significantly (*p* = 0.051). In the second analysis, which set the disease onset at 12 weeks of modelling after observing a significant increase in CVF at this time point, the AUCs of the ECV, native T1, and LVEF were 0.981 (95% CI, 0.969–0.990), 0.882 (95% CI, 0.855–0.910), and 0.857 (0.801–0.906), respectively (Fig. 5b). Here, the AUC of the ECV was found to differ significantly from that of the EF (*p < *0.001) and native T1 (*p* < 0.001); again, the AUCs of the native T1 and EF did not differ significantly (*p* = 0.821).

## Discussion

The present study aimed to assess the diagnostic feasibility of T1-mapping CMR for the evaluation of early myocardial changes in a rabbit model of doxorubicin-induced cardiotoxicity. Notably, in this model, the ECV changed significantly beginning at week 3 of modelling, or earlier than the observed changes in the native T1 and LVEF. Accordingly, the ECV had a higher diagnostic value than either the native T1 value or LVEF, and correlated well with the histology findings.

Doxorubicin, a representative anthracycline, is highly effective against a broad spectrum of malignancies, including lymphoma, sarcoma, and breast cancer, as well as other malignancies that occur in young female patients^4, 12^. Given its status as the most commonly used chemotherapeutic agent in cancer patients and the increasing cancer survival rate, the clinical significance of anthracycline-induced cardiotoxicity is expanding^12^.

Anthracycline-induced cardiotoxicity is considered a continuum that begins with subclinical myocardial cell injury and, leads to an early asymptomatic decline in LVEF that can develop into symptomatic heart failure^13^. The early detection and treatment of cardiotoxicity is critical for the recovery of cardiac function and reduction in the incidence of associated adverse cardiac events^13, 14^. Although the need for cardiac monitoring of asymptomatic anthracycline-treated adult patients is generally acknowledged, the existing guidelines offer no clear consensus regarding the timing or duration of such surveillance^14, 15^.

Currently, LVEF identification is most commonly used to screen for cardiotoxicity. Although MUGA scans and echocardiography are widely used to monitor the EF in cancer patients^7–9, 16, 17^, these methods are associated with a low sensitivity and specificity^17^; in addition, functional approaches cannot detect subclinical cardiac damage before an apparent and irreversible decrease in cardiac function. Accordingly, additional strategies for the early detection of cardiac damage and monitoring of treatment-induced cardiotoxicity are needed^16^. In this light, T1 mapping has emerged as a promising quantitative method for the detection of subclinical myocardial changes in various cardiomyopathies^18, 19^. Furthermore, T1 mapping and ECV measurement have been considered promising tools for the detection and quantification of myocardial damage resulting from chemotherapy-induced cardiotoxicity^10, 11^.

In our study, we observed changes in myocardial ECV without detectable changes in LVEF in early models; specifically, the ECV changed significantly at 3 weeks after model induction, whereas significant changes in the LVEF and native T1 were not observed until after 6 weeks. Furthermore, the mean CVFs of the LV were significantly higher than the baseline at 12 weeks. Small differences in native T1 between the normal and abnormal myocardium may be a cause of a later elevation of native T1.

Anthracycline-induced chemotoxicity results from the production of free radicals in both normal and malignant cells; these radicals react with oxygen to produce superoxide anion radicals, which damage the collagen network and cardiomyocytes^20, 21^. Previous studies demonstrated that at the tissue level, early anthracycline toxicity is associated with myocardial inflammation, vacuolization, cell swelling, and oedema, and that these changes occur before the onset of myocardial dysfunction^22, 23^. Our study also demonstrated tissue changes in early anthracycline toxicity models (i.e., week 3), particularly degenerative myocardial changes, vacuolization, and interstitial oedema. Notably, all of these types of damage, other than fibrosis, would cause an increase in the ECV in early models^20, 23^. The process of myocardial injury, which was quantitatively evaluated using Billingham scores, was also found to begin at week 3 along with the elevation of the ECV, thus corresponding to the subclinical stage before the LVEF and CVF changed significantly. In other words, myocardial injury preceded the development of fibrosis, and the myocardial ECV might reflect subclinical myocardial injury resulting from anthracycline-induced cardiotoxicity. An ECV based on a T1 mapping sequence could serve as an early marker of myocardial damage.

In our study, the CVF was higher in the RV insertion sites than in the LV throughout the modelling period. The RV and right atria are known to have significantly higher collagen concentrations than the LV and left atria^24, 25^. A previous study reported that because of this higher collagen concentration, the RV has a significantly higher myocardial native T1 value than the LV^24^. Notably, the RV and LV myocardium are combined at the RV insertion site, and accordingly this site had a higher CVF when compared with other parts of the myocardium. Our results also demonstrated that the papillary muscle had the highest ECV among all LV areas. An increase in the amount of abnormal collagen in the papillary muscle in response to chemotherapy-induced cardiotoxicity would greatly impair the contractile capacity and electrical conductance, thus severely compromising cardiac function^26^. However, even analyses of RV insertion sites and papillary muscles are not critical to a diagnosis of cardiotoxicity. Such a diagnosis might be facilitated by an additional focus on subclinical cardiotoxicity in cases without apparent myocardial fibrosis.

The strengths of our study included the availability of serial data based on the modeling time and a histologic evaluation of the myocardial structure. Our results also demonstrated that T1-mapping CMR could detect early myocardial damage.

Additionally, native T1 and the ECV might facilitate the detection of anthracycline responders before LV functional impairment becomes evident, and thus allow therapeutic adjustments and consideration of other treatment regimens. Further studies are required.

We also note several limitations of our study. First, the sample size was small, and our inclusion of extreme injury models led to a high incidence of severe cardiotoxicity, which could be considered a potential weakness. Further study is required to evaluate the incidence of cardiotoxicity in patients who exhibit early increases in ECV while receiving clinical doses of chemotherapy, as well as the prognostic value of an early increase of ECV in the clinical patient group.

Second, in the statistical analysis, we did not correct the significance level (e.g., Bonferroni adjustment) for multiple comparisons in the post hoc analysis. Some results with p-values near 5% might therefore be false positives. A further confirmatory study involving a larger sample size is needed to confirm the ability of our method to detect anthracycline-induced cardiotoxicity. Third, the small size of the papillary muscle precluded an evaluation of CVF. Finally, the control group was limited and did not include animals subjected to placebo treatment; rather, this group comprised all animals that underwent pre-modelling CMR. However, the use of the same animals in both groups allowed us to compare the baseline and serial data from each modelling time point in the same subject.

In conclusion, myocardial T1 mapping is a reliable and non-invasive method for the detection and serial monitoring of cardiotoxicity. This technique could form the basis of an appropriate strategy for the early detection of subclinical cardiotoxicity, even before a conventional critical doxorubicin dose has been reached.

## Methods

### Animal model and drug administration

All experiments were approved by animal care and use committee (IACUC) of Yonsei University Health System (approval number: 2014–0388) and were performed according to National Institutes of Health guidelines^27^. Twenty rabbits (adult male New Zealand White; body weight: 3**–**4 kg) were included in this study. Three rabbits were sacrificed after initial scanning to provide baseline histological findings. The remaining 17 rabbits received 1.0-mg/kg injections of doxorubicin (doxorubicin hydrochloride; Cayman Chemical, Ann Arbor, MI, USA) twice weekly until the time of sacrifice for histological evaluation. We followed a protocol that involved the lowest mortality rate in our previous study^28^. Although doxorubicin is known to be particularly venotoxic^29^, we did not experience difficulties with preserving vein integrity and did not encounter problems such as vein or skin necrosis during the maximum study period of 16 weeks.

### Animal preparation before CMR examination

Rabbits underwent MR examinations prior to drug administration to provide baseline data, followed by MR examinations 3 weeks after the first doxorubicin injection and every 2 weeks thereafter until the end of the specific post-modelling time period. Before each MR examination, rabbits were anesthetized with an intra-muscular injection of tiletamine (30 mg/kg, Zoletil; Vibac Laboratories, Carros, France) and xylazine (5 mg/kg, Rompun; Bayer, Seoul, Korea). The auricular veins of each rabbit were prepared for contrast injection. Venous sampling was performed to determine the haematocrit (Hct) values of all rabbits immediately before the MR examinations. After anaesthesia induction, the animals were intubated and mechanically ventilated (Mekant, MEKICS, Seoul, Korea) using a mixture of oxygen and isoflurane.

### CMR protocol

CMR was performed using a 3-T MR scanner (Magnetom Trio Tim; Siemens Healthcare, Erlangen, Germany) with a six-channel anterior body matrix coil and posterior part of a 12-channel head matrix coil. Cardiac localization was achieved using a steady-state free-precession sequence under electrocardiogram (ECG) gating. Pre- and post-contrast T1 mapping, cine, and late gadolinium enhancement (LGE) images were acquired. T1 mapping was performed using a prototype modified Look–Locker inversion recovery (MOLLI) sequence during the end-expiratory period in the mid-ventricular short-axis view. Pre-contrast T1 mapping images were acquired before contrast injection. Post-contrast T1 mapping images were acquired 13 min after the intravenous injection of a 0.2-mmol/kg dose of gadolinium contrast agent (Omniscan^®^; GE Healthcare, Princeton, NJ, USA). LGE MR imaging was obtained 15 min after contrast agent injection using a magnitude- and phase-sensitive inversion recovery-prepared steady-state free-precession sequence, with the inversion time adjusted to nullify the normal myocardium (please refer to the supplemental materials for the detailed CMR protocol).

### Image analysis

Two radiologists (Y.J.H., C.H.P.) with 10 years of experience in cardiovascular image interpretation analysed all MR images.

#### Functional MR image analysis

All MR cine images were transferred to cvi42 image analysis software (Circle Cardiovascular Imaging Inc., Calgary, AB, Canada). The LV function was assessed on short-axis cine MR images according to Simpson’s method. The endocardial and epicardial borders of the LV wall were delineated semi-automatically on end-diastolic and end-systolic images. The LV end-diastolic volume and LV end-systolic volume were automatically measured, and the LV ejection fraction (LVEF, %) and LV mass/weight (g/kg) were calculated.

#### CMR image analysis for the measurement of T1 and ECV fraction (%)

**Figure 6:**
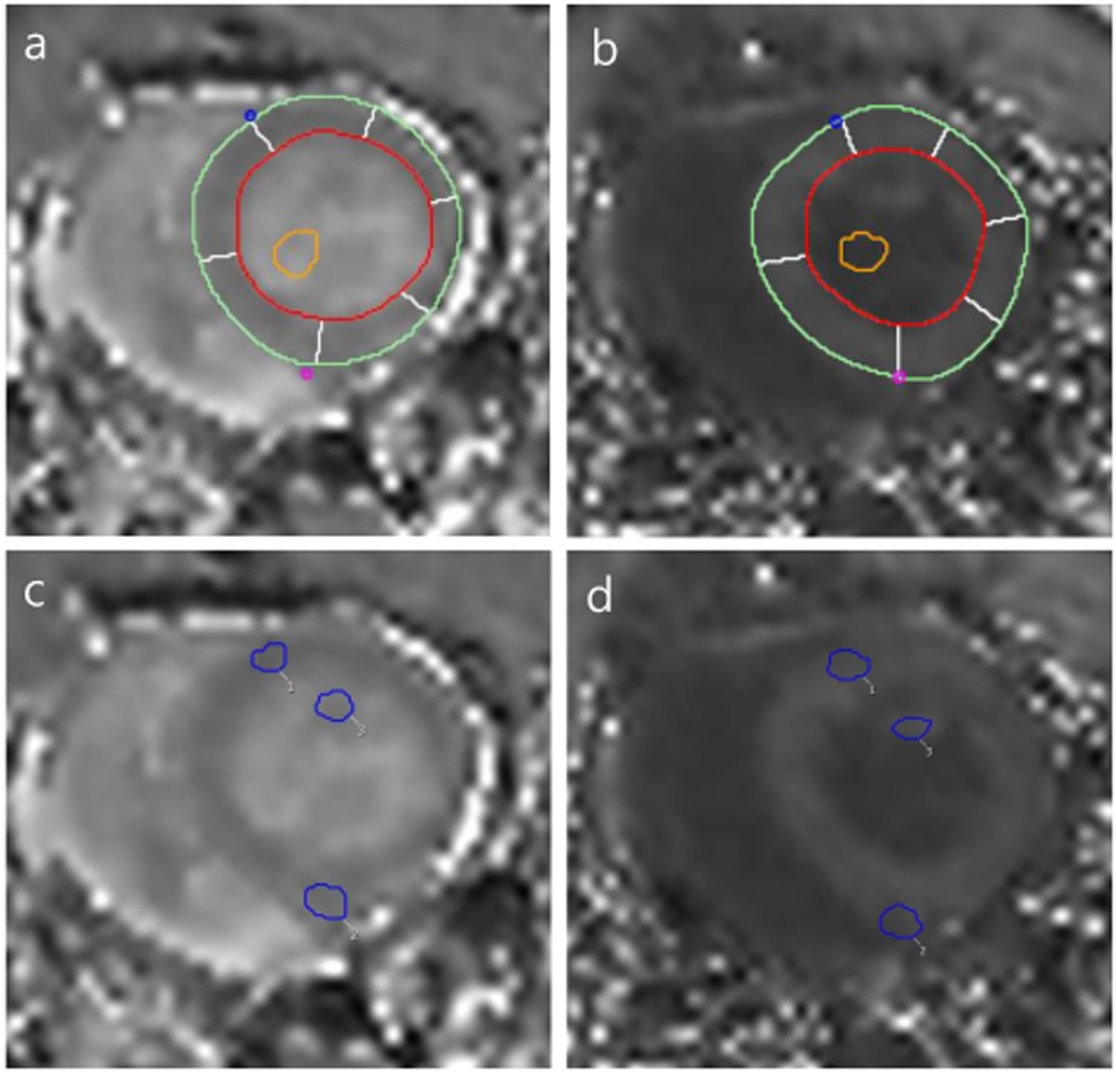
Measurement of T1 values in the myocardium and specific lesions of the left ventricle (LV) at the mid-ventricle on the short-axis plane. Endocardial (red line) and epicardial borders (green line) of the LV wall were delineated semi-automatically; six segments were delineated automatically (white lines). A round <5 mm^2^ region of interest (ROI; orange circle) was drawn in the LV cavity on pre-contrast (**a**) and post-contrast T1 mapping images (**b**). To measure T1 values of specific LV lesions in the short-axis plane, ROIs covering specific lesions (blue circle 1: right ventricle (RV) upper insertion site, 2: RV lower insertion site, 3: papillary muscle) were drawn on pre-contrast (**c**) and post-contrast T1-mapping images (**d**).

All MR pre- and post-contrast T1-mapping images were transferred to cvi42 image analysis software. For a regional analysis of the LV myocardium, the endocardial and epicardial borders of the LV wall were delineated semi-automatically on pre***-***and post***-***contrast T1 images obtained in a short-axis plane at the mid-LV level and divided into six segments based on AHA recommendations. A round, <5 mm^2^ region of interest (ROI) that avoided the papillary muscle was drawn in the LV cavity (Fig. 6a,b). Pre- (native) and post-contrast T1 values of the LV and blood cavity were measured automatically. The myocardial ECV was also calculated automatically using the Hct value and the native and post***-***contrast T1 values of the LV myocardium and blood cavity, as follows:

$$\begin{array}{rcl}{\bf{ECV}} & = & [({\bf{1}}/{\bf{T}}{{\bf{1}}}_{{\bf{post}}{\boldsymbol{-}}{\bf{contrastmyocardium}}})-({\bf{1}}/{\bf{T}}{{\bf{1}}}_{{\bf{pre}}{\boldsymbol{-}}{\bf{contrastmyocardium}}})]\\  &  & /[({\bf{1}}/{\bf{T}}{{\bf{1}}}_{{\bf{post}}{\boldsymbol{-}}{\bf{contrastblood}}})-({\bf{1}}/{\bf{T}}{{\bf{1}}}_{{\bf{pre}}-{\bf{contrastblood}}})]\times ({\bf{1}}\,{\boldsymbol{-}}\,{\bf{H}}{\bf{c}}{\bf{t}})\end{array}$$

The mean native T1 and ECV values of each segment were used. The native T1 and ECV were also measured at specific LV lesions, upper/lower RV insertion sites, and the papillary muscle. ROIs covering specific lesions (2–3 mm^2^) were drawn, and the pre- and post-contrast T1 and ECV values of specific lesions and the LV cavity were measured automatically (Fig. 6c,d).

### Histological analysis

After undergoing a final post-model MR scan, each rabbit was euthanized via potassium chloride while in an unconscious state induced by tiletamine/xylazine (30/5 mg/kg IM). The heart was extracted immediately after euthanasia for histologic evaluation. Each heart was fixed in 10% phosphate-buffered paraformaldehyde. After 1 week of fixation, the heart was sectioned serially along the short-axis plane using a rabbit heart slicer (Zivic Instruments, Pittsburgh, PA, USA), and the entire section of the heart at the mid-ventricle level, wherein papillary muscles were visible, was embedded in paraffin. Four-micrometre-thick slices of this section were obtained, stained with haematoxylin and eosin (H&E) and picrosirius red, and subsequently examined by a pathologist (H.S.P) blinded to the MR results.

#### Myocardial injury analysis

H&E staining was used to evaluate myocardial injury. The degree of myocardial injury was evaluated using a light microscope (optical BX 53 microscope; Olympus, Tokyo, Japan) and scored according to a seven-point scale described by Billingham *et al*.^4, 22^. Twelve sets of LV myocardium micrographs of (three each of the septum, lateral, superior, and inferior wall) were analysed per animal. The grading system was as follows: 0 = no damage; 0.5 = not completely normal; 1, 1.5, 2, 2.5, and 3 = damage to 1–5%, 6–15%, 16–25%, 26–35% and >35% of all cells, respectively.

#### Collagen volume fraction (CVF, %) analysis

Picrosirius red staining was used to evaluate the CVF in the extracellular space. Twelve sets of LV myocardium micrographs (three each of the septum, lateral, superior, and inferior wall; magnification, ×200) and six fields from the RV upper and lower insertion sites (magnification, ×400) were acquired per animal and transferred to a computer for further analysis. The mean percentage of fibrosis was also determined. Perivascular fibrosis was not included in this analysis^18^ (please refer to the supplemental materials for a detailed protocol).

### Statistical analysis

All continuous data are expressed as means ± standard deviations, and categorical variables are presented as numbers or percentages. The Shapiro–Wilk test was performed to evaluate data distributions.

A linear mixed model with restricted maximum-likelihood estimation was used to evaluate the time point of each variable (Hct, LV mass, LV mass/kg, EF, CVF, myocardial injury score) and the native T1 and ECV values of the whole LV and specific LV lesions (RV insertion sites, papillary muscles) in each experimental group that exhibited a significant change from the baseline group according to the modelling time. The MIXED protocol in SAS (version 9.2; SAS Institute, Cary, NC, USA) was used to generate this linear mixed model, which included fixed effects for time and specific lesions, interactions between time and specific lesions, and random intercepts for each animal. Time was considered a categorical variable in this model, and equal covariance was assumed between all-time points.

To compare the diagnostic performances of ECV, native T1, and LVEF, a ROC curve was constructed^30^. Post-modelling groups were considered positive for disease. A bootstrap method with 1,000 replications was used for an evaluation that yielded a 95% confidence interval (CI) and *p* value of the AUC. Spearman’s correlation was used to compare the ECV values of the LV myocardium and RV insertion site (mean ECV of the RV upper and lower insertion sites), as well as the myocardial injury scores and histologic CVFs. The CVFs of the LV myocardium and RV insertion site were compared using a paired t test. Inter-observer agreement regarding the measured native T1 and ECV values of the LV and specific lesions was assessed using ICCs, as well as a two-way random effects model that included the observer and animal as random effects. All statistical analyses were performed using SAS, and a *p* value < 0.05 was considered statistically significant.

## Electronic supplementary material

Supplementary Information
