# Author Correction: Early Detection and Serial Monitoring of Anthracycline-Induced Cardiotoxicity Using T1-mapping Cardiac Magnetic Resonance Imaging: An Animal Study

**DOI:** 10.1038/s41598-022-13822-w

**Published:** 2022-06-09

**Authors:** Yoo Jin Hong, Heae Surng Park, Kyunghwa Han, Chul Hwan Park, Tai Kyung Kim, Sae Jong Yoo, Ji Yeon Lee, Pan Ki Kim, Jin Hur, Hye-Jeong Lee, Young Jin Kim, Young Joo Suh, Mun Young Paek, Byoung Wook Choi

**Affiliations:** 1 Department of Radiology and Research Institute of Radiological Science, Severance Hospital, Yonsei University Medical Centre, Seoul, South Korea; 2 Department of Pathology, Gangnam Severance Hospital, Yonsei University College of Medicine, Seoul, South Korea; 3 Department of Radiology and Research Institute of Radiological Science, Gangnam Severance Hospital, Yonsei University Medical Centre, Seoul, South Korea; 4 Department of Veterinary Surgery, College of Veterinary Medicine, Konkuk University, Seoul, South Korea; 5 Siemens Ltd., Seoul, South Korea

Correction to: *Scientific Reports* https://doi.org/10.1038/s41598-017-02627-x, published online 01 June 2017

This Article contains an error. Jeffery Kihyun Park was incorrectly listed as an author, and all the authors, including Jeffrey Kiyhun Park, have agreed they should be removed due to insufficient contribution.

A corrected version of the Author list appears below.

“Yoo Jin Hong, Heae Surng Park, Kyunghwa Han, Chul Hwan Park, Tai Kyung Kim, Sae Jong Yoo, Ji Yeon Lee, Pan Ki Kim, Jin Hur, Hye-Jeong Lee, Young Jin Kim, Young Joo Suh, Mun Young Paek & Byoung Wook Choi”

In addition, the Author Contributions section,

“Y.J. Hong, B.W. Choi conceived experiment wrote the manuscript. Y.J. Hong, T.K. Kim, S.J. Yoo, C.H. Park, J. Park, P.K. Kim, J.Y. Lee conducted experiment. P.K. Kim, M.Y. Paek set up the CMR protocol. K. Han conducted statistical analysis. H.S. Park conducted pathological results analysis. J. Hur, Y.J. Kim H.Y. Lee, Y.J. Suh edited the manuscript.”

should read:

“Y.J. Hong, B.W. Choi conceived experiment wrote the manuscript. Y.J. Hong, T.K. Kim, S.J. Yoo, C.H. Park, P.K. Kim, J.Y. Lee conducted experiment. P.K. Kim, M.Y. Paek set up the CMR protocol. K. Han conducted statistical analysis. H.S. Park conducted pathological results analysis. J. Hur, Y.J. Kim H.Y. Lee, Y.J. Suh edited the manuscript.”

