# Supplementary material for: Early Detection and Serial Monitoring of Anthracycline-Induced Cardiotoxicity Using T1-mapping Cardiac Magnetic Resonance Imaging: An Animal Study
Source: Sci Rep. 2017 Jun 1;7:2663. doi: 10.1038/s41598-017-02627-x (PMC5453985; doi:10.1038/s41598-017-02627-x)
Supplement: Supplementary file 1 — Supplementary Information [file 41598_2017_2627_MOESM1_ESM.doc]

**Early Detection and Serial Monitoring of Anthracycline-Induced Cardiotoxicity Using T1-mapping Cardiac Magnetic Resonance Imaging: An Animal Study**

Dr Yoo Jin Hong^1^, Dr Heae Surng Park^2^, Mr Jeffrey Kihyun Park^1^, Dr Kyunghwa Han^1^, Dr Chul Hwan Park^3^, Ms Tai Kyung Kim^4^, Mr Sae Jong Yoo^4^, Ms Ji Yeon Lee^1^, Dr Pan Ki Kim^1^, Dr Jin Hur, MD^1^, Dr Hye-Jeong Lee^1^, Dr Young Jin Kim^1^, Dr Young Joo Suh^1^, Ms Mun Young Paek^5^, Dr Byoung Wook Choi^1^,*

^1^Department of Radiology and Research Institute of Radiological Science, Severance Hospital, Yonsei University Medical Centre, Seoul, South Korea

^2^Department of Pathology, Gangnam Severance Hospital, Yonsei University College of Medicine, Seoul, South Korea

^3^Department of Radiology and Research Institute of Radiological Science, Gangnam Severance Hospital, Yonsei University Medical Centre, Seoul, South Korea

^4^Department of Veterinary Surgery, College of Veterinary Medicine, Konkuk University, Seoul, South Korea

^5^Siemens Ltd., Seoul, South Korea

*Address for correspondence:

Byoung Wook Choi, M.D., Ph.D.

Department of Radiology, Research Institute of Radiological Science, Severance Hospital, Yonsei University College of Medicine, 50 Yonsei-ro, Seodaemun-gu, Seoul 120-752, South Korea

Tel: 82-2-2228-7400

Fax: 82-2-393-3035

E-mail: bchoi@yuhs.ac

**Supplement material**

**CMR protocol**

We have followed CMR techniques, which involved optimal image sequences based on the previous study. Cine images were acquired using a TrueFISP sequence in the short-axis plane with the following parameters: repetition time (TR)=3.9 ms, echo time (TE)=1.68 ms, flip angle=50°, phase number=25, slice thickness=3 mm, slice gap=0.6 mm, acquisition matrix=160×160, and field of view=150 mm×135 mm. For MOLLI imaging, three nonselective inversion pulses and a steady-state free-precession single-shot readout in the mid-diastolic phase were employed using the following parameters: field of view=150 mm×121 mm, acquisition matrix=192×155, slice thickness=3.5 mm, TR=3.6 ms, TE=1.45 ms, minimum inversion time = 105 ms, inversion time increment=60 ms, flip angle=35°, and Generalized Autocalibrating Partially Parallel Acquisitions (GRAPPA) with an acceleration factor of 2. Four images were acquired after the first inversion pulse, followed by a pause of six heart beats, after which four images were acquired after the second inversion pulse, followed by a pause of six heart beats. Finally, six images were acquired after a third inversion pulse. Fully automated non-rigid motion correction was used to register the individual TI images prior to inline T1fitting with a mono-exponential three-parameter fit. LGE images were obtained along the same axial plane and at the same slice thickness used for the T1 mapping images (TR=3.3 ms, TE=1.41 ms, field of view=200 mm×162 mm, thickness=3.5 mm, matrix=224 × 201, non-selective inversion recovery, flip angle=70º, bandwidth=770 Hz/pixel, number of trigger pulses=2).

**Collagen volume fraction (CVF, %) analysis**

All histopathological image analyses were conducted using ImageJ software (Ver. 1.48v; National Institutes of Health, Bethesda, MD, USA). First, the image format was converted from a 16-bit signed Digital Imaging and Communications in Medicine file to an 8-bit unsigned binary gray-scaled Tagged Image File Format to simplify the image information. Second, a histogram of the histological image was generated for thresholding segmentation, using the internal histogram function in the ImageJ software package. Third, two-point thresholding segmentation was performed to separate fibrosis from myocardial cells on the images. Normally, on an 8-bit binary image, fibrosis signal intensity ranges from approximately 0 to 30, and myocardial cell intensity ranges from 200 to 255. Two thresholds, located at the 40- and 180-pixel intensity points, were used to estimate both the fibrosis and myocardial cell areas, allowing an evaluation of the image fibrosis to myocardium ratio (percentage).
